# Post-authorisation experience and reported adverse events following use of a virus-like particle chikungunya vaccine, United States and Germany, up to August 2025

**DOI:** 10.2807/1560-7917.ES.2025.30.44.2500792

**Published:** 2025-11-06

**Authors:** Benedetto Simone, Florian Lienert

**Affiliations:** 1 Bavarian Nordic Berna GmbH, Thoerishaus, Switzerland; 2 Bavarian Nordic A/S, Hellerup, Denmark

**Keywords:** Chikungunya virus, vaccination, virus-like particles, pharmacovigilance

## Abstract

Older adults are at increased risk of severe chikungunya. Some countries advise against vaccinating ≥ 60 or ≥ 65-year-olds with the licenced live-attenuated vaccine (CHIKV LA, IXCHIQ), following severe adverse event (SAE) reports. A virus-like particle vaccine (CHIKV VLP, VIMKUNYA) is licensed in the United States (US), EU/EEA and the United Kingdom. Up to 31 August 2025, over 12,500 doses were administered in US and Germany; no SAEs in ≥ 65-year-olds were reported. Early post-authorisation data support its favourable safety profile in ≥ 65-year-olds.

Two chikungunya vaccines are currently licensed in parts of the world, including the European Union/European Economic Area (EU/EEA): IXCHIQ (CHIKV LA, Valneva), a live-attenuated vaccine (LA) [1]; and VIMKUNYA (CHIKV VLP, Bavarian Nordic), a virus-like particle (VLP) vaccine [2]. Following reports of severe adverse events (SAEs) in older adults vaccinated with CHIKV LA in early 2025 in the US and France [3,4], national authorities from several countries introduced age restrictions to the use of this vaccine. We report here early post-authorisation surveillance data for CHIKV VLP from the United States (US) and Germany, the first countries to introduce the vaccine, up to 31 August 2025, with a focus on the use in ≥ 65-year-olds. Official recommendations to vaccinate against chikungunya disease in both countries focus on individuals travelling to countries or territories at risk of transmission, and on laboratory workers with potential for exposure to chikungunya virus [4,5]. 

## Resurgence of chikungunya and relevance of vaccinating older adults

Chikungunya is a viral illness marked by sudden onset of fever and severe joint pain, which can persist for months to years [6]. A resurgence of chikungunya virus (CHIKV) circulation in 2024–25 led to large outbreaks documented across South Asia, the Americas and regions of Africa [7]. Substantial autochthonous transmission also occurred in southern Europe in 2025 [8]. Chikungunya poses a particular risk to older adults (≥ 65 years), who are more likely to experience severe and long-lasting complications [9,10]. During the recent outbreak in La Réunion (France) in 2024–25, older adults represented almost half of all chikungunya hospitalisations (146/340), and almost two-thirds of severe cases (36/55; defined as those with at least one organ failure) [11]. A meta-analysis of literature found that age ≥ 60 years is a risk factor for mortality in chikungunya (odds ratio: 19.5; 95% confidence interval: 2.0–191.9) [12]. Considerations for vaccination in older adults living in areas at risk of chikungunya transmission, or visiting such areas, are therefore of high relevance. 

## CHIKV VLP adverse event reports and doses sold/administered

On 30 September 2025, we searched for adverse events (AE) reported following administration of CHIKV VLP in the US and Germany up to 31 August 2025 in the Vaccines Adverse Event Reporting System (VAERS) and Eudravigilance, the pharmacovigilance databases of the US and EU/EEA, respectively. 

We collected US in-market sales data of CHIKV VLP, following the same approach used in by Hills et al. in an assessment of post-authorisation safety for CHIKV LA [13]. Available national CHIKV VLP data from the launch in March 2025, i.e. the point at which the vaccine became available to purchase, up to 31 August 2025 were purchased from IQVIA, a company that provides healthcare data [14,15]. We collected information on the number of vaccine recipients aged ≥ 65 years where available, i.e. through sales data from retail pharmacies.

In Germany, where chikungunya has never been reported [6], vaccination is recommended exclusively to travellers to at-risk areas [5]. Because travel vaccines are often reimbursed by private insurances, we were able to purchase national data from IQVIA on numbers of vaccine doses administered from the launch in May 2025, up to 31 August 2025.

## Post-authorisation experience in the United States

A total of 9,346 CHIKV VLP doses were sold in the US. Based on available data from retail pharmacies, we estimated that 46% of doses were administered to individuals aged ≥ 65 years (n = 4,300 doses).

No serious AEs (SAEs), hospitalisations or deaths were reported in VAERS in ≥ 65-year-olds, or overall. Four non-serious (ns) AEs in three individuals were reported: ns-AE1 was a syncope (fainting) in an adolescent following co-administration of Ixiaro (Japanese encephalitis vaccine, inactivated, adsorbed, Valneva [16]; right shoulder) and CHIKV VLP (left shoulder): the episode lasted less than one minute and the patient *“was given soda and water”*; ns-AE2 and ns-AE3 were erythema and warmth in the left shoulder: the patient was prescribed cephalexin and cetirizine; and for ns-AE4, the event was described as ‘product preparation error’ and not specified further.

## Post-authorisation experience in Germany

A total of 3,470 CHIKV VLP doses were administered. We were not able to collect information on how many doses were administered to older adults. One SAE, an atrial fibrillation, was reported in an adult aged 18–64 years. The event is reported to have lasted 0.0 days and the patient to have recovered. Three additional non-serious AEs were reported: ns-AE5 was limb discomfort, parosmia, pyrexia and taste disorder in an 18–64-year-old person; ns-AE6 comprised arthralgia, asthenia, back pain, burning sensation, fatigue, headache, musculoskeletal stiffness and myalgia in an 18–64-year-old person; and ns-AE7 included arthralgia, muscle twitching, pain in extremity and paraesthesia in a 65–84-year-old person. Concomitant vaccinations were not reported for any of these AEs.

## Discussion

This report presents early available data for the post-authorisation use of CHIKV VLP, a virus-like particle vaccine (VIMKUNYA). For the two countries where most data are available, the US and Germany, up to 31 August in the population aged ≥ 65 years, there were no SAEs, hospitalisations or deaths reported following administration of the vaccine. Only one SAE was reported in Germany in an adult (18–64 years). Importantly, we did not identify any of the neurological SAEs that were reported following administration of the licenced CHIKV LA (IXCHIQ) in the population aged ≥ 65 years [13].

 National technical recommending bodies in Canada, Germany, France and United Kingdom (UK), among others, currently recommend against using CHIKV LA in people ≥ 65 years. The vaccine requires careful risk benefit assessment for use in individuals with age-related immune decline. [5,17-19]. In response to an outbreak in La Réunion, a French Overseas Department, in February 2025, French Health Authorities issued a vaccination recommendation for local older adults to receive the live-attenuated vaccine; this recommendation was revised in April 2025 following a risk re-assessment triggered by the report of a fatal AE in an 84-year-old [3,20]. In the US, use of CHIKV LA has been suspended since August 2025 [4]. 

An analysis [13] conducted in late spring 2025 by the Centers for Disease Control and Prevention (CDC) and the Food and Drug Administration (FDA) in the US estimated 2024 rates of SAEs and hospitalisations following administration of the CHIKV LA among ≥ 65-year-olds to be 82 per 100,000 and 68 per 100,000, respectively. These estimates were based on sales data obtained from IQVIA. These data are important to complement the clinical trial experience for the vaccine: in a pivotal phase 3 study, 3,082 participants had received the vaccine, including 346 aged ≥ 65 years, and two vaccine-related SAEs were reported [21]. 

The CHIKV VLP vaccine is indicated in individuals aged ≥ 12 years, and was licensed in February 2025 in the US and the EU/EEA, and in May 2025 in the UK [2,22,23]. The VLPs contained in the vaccine do not contain genetic material and are not able to replicate or infect cells [24]. Current national recommendations do not restrict the use of the vaccine in older adults [4,5,18,19]. A phase 3 clinical study conducted in ≥ 65-year-olds showed no notable differences in AE rates between groups (vaccine n = 206; placebo n = 207), with most AEs being grade 1 or 2 in severity and of short duration; four (2%) of vaccine recipients and three (1%) in the placebo group reported a SAE, none of which was vaccine-related; no deaths occurred [24].

Up to 31 August 2025, the vaccine had only been available in France and UK for a short period of time and we did not collect vaccine administration data. There were no AE reports from France in Eudravigilance or from the UK in the Yellow Card data portal. Austria, Denmark, Finland, Italy, Norway, Portugal, Spain and Sweden introduced the vaccine after 31 August 2025 and are, therefore, not covered in this report. Additional countries will follow suit.

Early post-authorisation observations appear consistent with the findings from the pivotal clinical trial for CHIKV VLP in the population aged ≥ 65 years [24]. The data presented here provide early insights on the use of the CHIKV VLP vaccine in a real-world setting. 

The frequency of reported AEs in early post-authorisation settings also aligns with experience from other marketed VLP-based vaccines, such as those against hepatitis B and human papillomavirus, which have been used and studied extensively for decades [25].

This report has several limitations. Not all the doses of vaccine sold in the US would have been administered at the time this analysis was conducted. Furthermore, administration data were available only from some retail pharmacies, which cover a proportion of all the vaccine doses administered. It is fair to assume, however, that a substantial proportion of CHIKV VLP doses would have been given to older adults overall, given that, from May to August 2025, the use of CHIKV LA was paused in ≥ 60-year-olds [4], and travellers of this age group are broadly understood to be at increased risk of severe chikungunya. On the contrary, the data presented from Germany are doses administered: these data reflect prescriptions from a proportion of private health insurance providers, and it is likely an underestimation of the actual number of administered doses. While the number of doses administered in older adults is not available, it is reasonable to assume that it is a substantial proportion, given that CHIKV LA is not recommended in the population aged ≥ 60 years in Germany [12].

## Conclusions

Continued surveillance will be essential as the CHIKV VLP vaccine use expands, and the work done by regulatory authorities in detecting, investigating and characterising SAEs, including – potentially – very rare ones, remains critical. The early post-authorisation data corroborate the safety findings from clinical studies for CHIKV VLP in  individuals ≥ 65 years old. This evidence will be valuable for policymakers and healthcare providers when assessing risks and benefits of vaccinating older adults at risk of chikungunya exposure.

## Data Availability

Data from AE reports to VAERS (https://vaers.hhs.gov/data.html), Eudravigilance (https://www.adrreports.eu/en/search_subst.html) and the Yellow Card data portal (https://yellowcard.mhra.gov.uk) are publicly available.
